# WSNs data acquisition by combining expected network coverage and clustered compressed sensing

**DOI:** 10.1371/journal.pone.0326078

**Published:** 2025-06-17

**Authors:** Zhouzhou Liu, Yangmei Zhang, Yang Bi, Jingxuan Wang, Yuanyuan Hou, Chao Liu, Guangyi Jiang, Shan Li

**Affiliations:** 1 School of Computer Science, Xihang University, Xi’an, China; 2 School of Electronic Engineering, Xihang University, Xi’an, China; Maulana Abul Kalam Azad University of Technology West Bengal, INDIA

## Abstract

To tackle the challenges of extensive data transmission and high redundancy in wireless sensor networks (WSNs), this study proposes a novel data collection scheme based on expected network coverage and clustered compressive sensing (CS). First, the K-medoids clustering algorithm organizes nodes within the WSN coverage area into clusters. Combined with an optimized network coverage algorithm, a node scheduling strategy is introduced to focus on critical observation areas while minimizing overall energy consumption. Next, by analyzing the relationship between network clustering and node deployment, a weakly correlated observation matrix is designed to mitigate the impact of packet loss on data collection. Finally, the sparrow search algorithm is employed to enhance the accuracy of CS data reconstruction at the cluster head. Simulation results demonstrate that, compared to existing data collection schemes, the proposed approach significantly reduces WSN transmission overhead, ensures accurate recovery of raw data, decreases data reconstruction error, and extends network lifetime.

## Introduction

With the rapid advancement of wireless network communication technology, wireless sensor networks (WSNs) have found extensive applications in environmental monitoring, tracking, positioning, smart buildings, and industrial control [1,2]. These networks are increasingly garnering attention both domestically and internationally due to their potential to transform how we collect and manage data [3,4]. However, current data collection processes in large-scale WSNs face several pressing challenges that directly contradict the core requirements of next-generation IoT systems demanding real-time responsiveness and decade-long battery life:

(1) **Energy-resource imbalance** caused by limited node computing power and uneven energy consumption, particularly in large-scale networks with dynamic coverage requirements.
(2) **Redundancy and inefficiency** in raw data transmission due to spatial-temporal correlations among sensors, which conventional compression techniques fail to address effectively [5].
(3) **Data reliability degradation** from packet loss on unreliable links, exacerbated by environmental interference and outdated error-correction mechanisms [6].

A well-designed network coverage strategy is fundamental to the effectiveness of WSN applications, as it directly affects the quality of data collection and processing outcomes. Recent advancements in non-uniform clustering protocols have evolved from static radius adjustment to dynamic multi-objective optimization. Wang et al. [7] proposed a Spider Wasp Optimizer that integrates residual energy, node density, and mobility patterns into cluster head selection, achieving longer network lifespan than traditional LEACH protocols. Concurrently, hierarchical coverage optimization has emerged as a dominant paradigm. Ou et al. [8] developed a Multi-Strategy Grey Wolf Optimizer (MS-GWO) incorporating Cauchy mutation for local minima escape, dynamic inertia weights balancing exploration-exploitation, and adaptive convergence factors, which improve coverage rates in irregular terrains. Meanwhile, compressive sensing (CS) has emerged as a critical technique for reducing data redundancy by exploiting spatial correlations [9–11]. Geon et al. [12] proposed an algorithm utilizing the sparse projection matrix for data collection. It employs the minimum cost sparse projection matrix to sample node data and applies the Hungarian algorithm for accurate data reconstruction. However, this algorithm does not adequately account for the impact of packet loss on data collection results and is suitable only for scenarios without packet loss. As networks continue to expand, reasonable network clustering becomes more advantageous for improving the data transmission management efficiency. Zhou Wei et al. [13] presented an airborne clustering data collection algorithm based on CS, considering the data collection technology under hardware resource constraints and providing valuable insights for practical engineering applications of WSNs. Meanwhile, Zhang et al. [14] introduced a similarity-based clustering algorithm, CDSW, and a data collection strategy based on data sensing and similarity, DSCDC. This approach aims to reduce the transmission of temporally and spatially redundant data, thereby extending the network’s lifetime. However, static network models neglect dynamic environmental factors, leading to suboptimal coverage in real-time monitoring scenarios. Adaptive CS frameworks now dynamically adjust sampling rates based on sparsity variations [15]. Rani et al. [16] introduced a hybrid CS-based data aggregation model that integrates a Golden Circle-Inspired Optimization (HGCIO) algorithm with a CNN-LSTM framework, balancing energy efficiency and computational accuracy. But CS-based methods often assume lossless channels, failing to address packet loss during compressed data transmission. And computational complexity escalates exponentially in large-scale networks, particularly when integrating coverage optimization with CS [17].

In practical applications, it is often essential to monitor specific key areas within the sensing range. The demand for real-time and highly accurate data collection methods is increasing. To address this need, this paper presents a pioneering data collection scheme for WSNs that integrates expected network coverage with cluster-based compressive sensing techniques. This approach is motivated by the growing necessity for real-time and reliable data collection tailored to specific key monitoring areas. By analyzing node coverage redundancy and the dynamics between network clustering and node deployment, we determine the optimal configuration for network clusters. Additionally, we develop a novel cluster compressive sensing data collection algorithm that utilizes a weak correlation observation matrix to enhance efficiency. Through comprehensive simulation experiments, we validate the effectiveness of our proposed method, demonstrating its potential to improve data collection accuracy and extend network lifespan. Overall, our contributions lines in:

- A dynamic coverage-clustering model that integrates non-uniform node deployment with real-time coverage adjustments using bio-inspired optimization;
- A robust cluster-based CS algorithm employing a weak-correlation observation matrix to tolerate packet loss while maintaining reconstruction accuracy.

### Problem description

**Fig 1:**
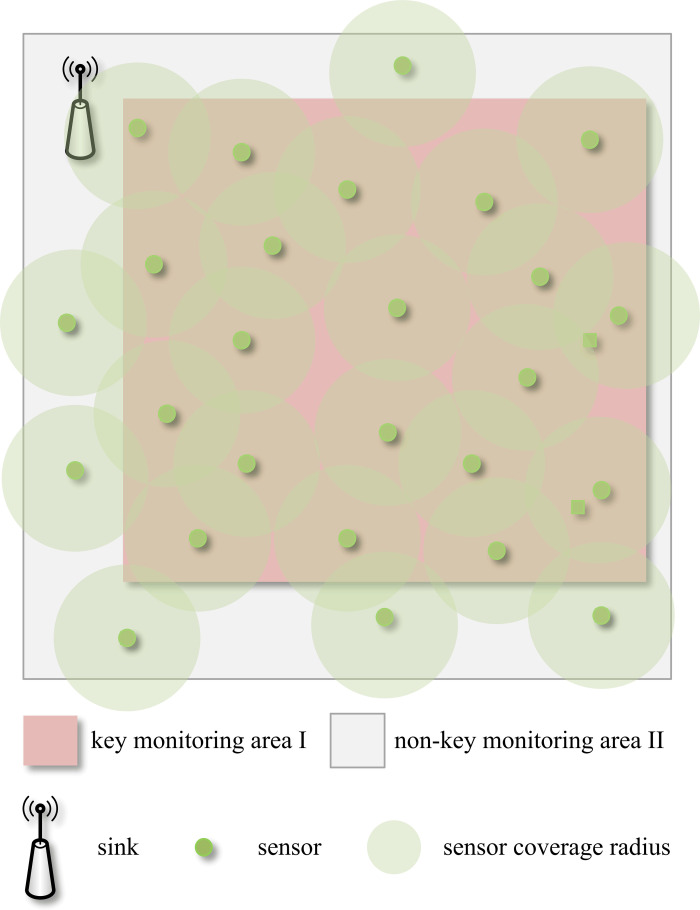
Monitoring Area.


Constructing a double square area as the monitoring area, as illustrated in Fig 1. This N×N area is segmented into two distinct sections: key monitoring area I and non-key monitoring area II. The entire monitoring area is further divided into smaller square units, totally N×N units. Q sensor nodes $V=\left\{SN_{i}\right\}$ (i=1,2,⋯,Q) and a sink node are deployed into the monitoring area. The sensor nodes adopt the energy consumption model from Reference 12 with initial energy heterogeneity. Both the sensor nodes and the sink node’s location information are presumed to be known. The data collected by node $SN_{i}$ is denoted by $f_{i}$, forming the sensor acquisition information vector $X_{Q\times 1}={\left(f_{1},f_{2},\cdots ,f_{Q}\right)}^{T}$. For K event sources within the monitoring area, denote them with the vector $G_{N^{2}\times 1}={\left(g_{1},\cdots ,g_{j},\cdots ,g_{N^{2}\times 1}\right)}^{T}$ (where $g_{j}$ stands for the signal strength of an event source in the j th square unit. A value of zero signifies the absence of an event source in that particular unit.), apparently there is

$$X_{Q\times 1}=\Psi_{Q\times N^{2}}G_{N^{2}\times 1}=\left[\begin{array}{ccc} {}*20c\psi_{11} & \cdots & \psi_{1N^{2}}\\ \vdots & \ddots & \vdots \\ \psi_{Q1} & \cdots & \psi_{QN^{2}} \end{array}\right]\left(\begin{array}{c} {}*20cg_{1}\\ \vdots \\ g_{N^{2}} \end{array}\right)$$ (1)

where $\Psi_{Q\times N^{2}}$ is the propagation matrix. Since the sink node usually receives and processes only parts of the sensor node information, the observation matrix $\Phi_{M\times Q}$ is selected for fusion processing on $X_{Q\times 1}$, i.e.,


$$y=\Phi_{M\times Q}X_{Q\times 1}=\Phi_{M\times Q}\Psi_{Q\times N^{2}}G_{N^{2}\times 1}$$ (2)

where *M* is the number of sensor node information processed by the sink node.

When WSNs are divided into C clusters according to certain criteria, the cluster head node $CH_{k}$ (k=1,2,⋯,C) uses the observation matrix $\Phi_{M\times Q_{k}}$ to fuse the data of $Q_{k}$ nodes within its cluster $\begin{array}{c} X_{Q_{k}\times 1}={\left(f_{1}^{\;k},f_{2}^{\;k},\cdots ,f_{Q_{k}}^{\;k}\right)}^{T} \end{array}$ to yield the measurement value $y_{M\times 1}$, i.e.,


$$\begin{array}{c} y=\left[\begin{array}{c} {}*20cy_{1}\\ y_{2}\\ \vdots \\ y_{M} \end{array}\right]=\Phi_{M\times Q_{k}}X_{Q_{k}\times 1}=\left[\begin{array}{ccc} {}*20c\phi_{11} & \cdots & \phi_{1Q_{k}}\\ \vdots & \ddots & \vdots \\ \phi_{M1} & \cdots & \phi_{MQ_{k}} \end{array}\right]\left[\begin{array}{c} {}*20cf_{1}^{k}\\ \vdots \\ f_{Q_{k}}^{k} \end{array}\right]\\ =\left[\begin{array}{ccc} {}*20c\phi_{11} & \cdots & \phi_{1Q_{k}}\\ \vdots & \ddots & \vdots \\ \phi_{M1} & \cdots & \phi_{MQ_{k}} \end{array}\right]\left[\begin{array}{ccc} {}*20c\psi_{11} & \cdots & \psi_{1N^{2}}\\ \vdots & \ddots & \vdots \\ \psi_{Q_{k}1} & \cdots & \psi_{Q_{k}N^{2}} \end{array}\right]\left(\begin{array}{c} {}*20cg_{1}\\ \vdots \\ g_{N^{2}} \end{array}\right)=\Phi_{M\times Q_{k}}\Psi_{Q_{k}\times N^{2}}G_{N^{2}\times 1} \end{array}$$ (3)

**Fig 2:**
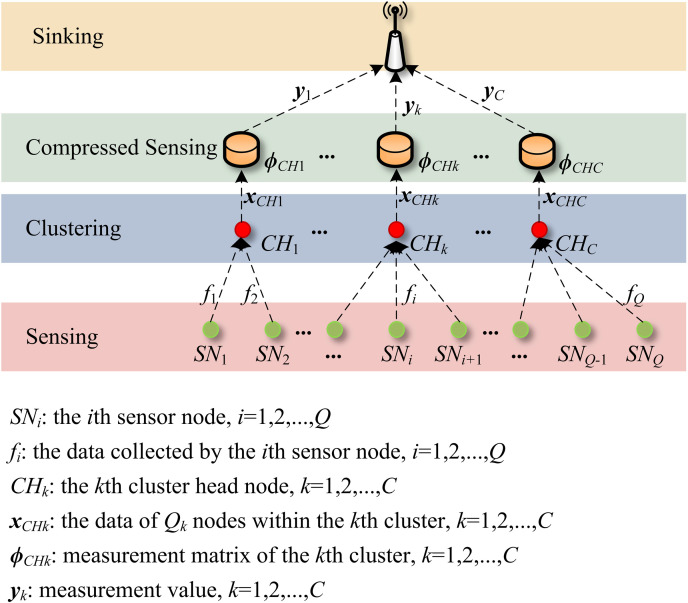
WSNs Data Collection Model.

Due to the number of event sources $K\ll N^{2}$, $G_{N^{2}\times 1}$ is a sparse vector and $\Psi_{Q_{k}\times N^{2}}$ is a sparse basis. At this point, equation (3) conforms to the CS model. When an appropriate measurement matrix $\Phi_{M\times Q_{k}}$ is selected, the sparse vector $G_{N^{2}\times 1}$ can be derived by $y_{M\times 1}$, which in turn yields the data collection information $X_{Q\times 1}={\left(f_{1},f_{2},\cdots ,f_{Q}\right)}^{T}$. The data collection process in WSNs is illustrated in Fig 2, facilitating a clearer overarching framework of our data collection scheme.

The application of clustered compressed sensing for data collection not only decreases the volume of data acquired by the sink node, but also guarantees high accuracy in data reconstruction through reasonable design of the observation matrix.

### Proposed scheme

**Fig 3:**
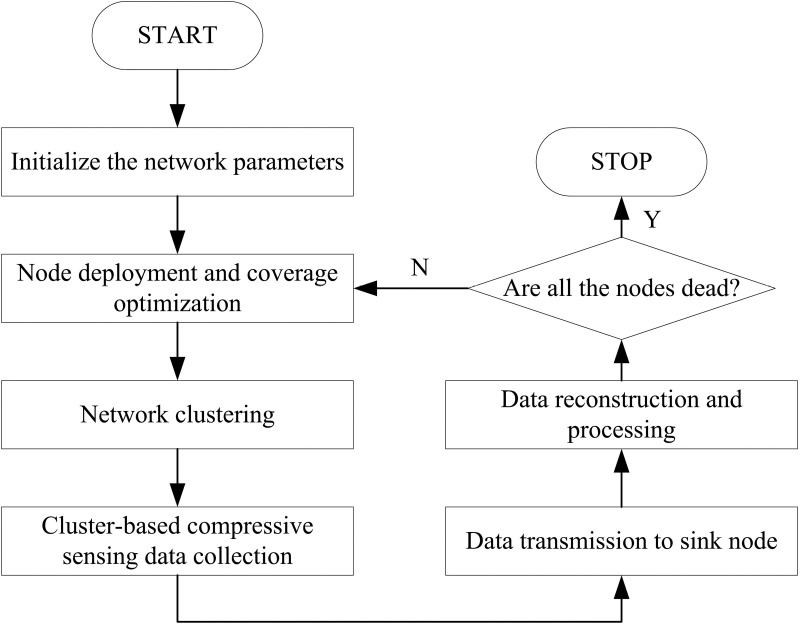
Flowchart of the proposed scheme.

The workflow of the proposed scheme is systematically illustrated in Fig 3. Firstly, the network parameters are initialized. Then, an energy-efficient coverage optimization is implemented through selective node activation, effectively balancing network coverage with energy conservation requirements. Following this optimization, the network architecture is strategically reconfigured by partitioning the nodes into multiple clusters. Each cluster is designed with an optimal intra-cluster communication hop distance to minimize signal attenuation and energy consumption during data transmission. Upon successful cluster formation, cluster heads systematically aggregate and process data from member nodes using custom-designed measurement matrices. This compression process enables efficient data projection, significantly reducing transmission payload. The compressed data packets are then relayed through multi-hop communication paths to the centralized sink node. The data reconstruction begins when the sink node receives the compressed datasets. To ensure data integrity, an error correction mechanism employing a weak correlation matrix is activated to detect and rectify potential transmission-induced distortions. Ultimately, the compressed signals undergo precise reconstruction through the Sparrow Search Algorithm (SSA), which effectively decomposes and recovers the original signal components.

### Expected network coverage optimization

The objective of optimizing network coverage is to effectively utilize the minimal number of sensor nodes required to cover the monitoring area while meeting the designated coverage requirements. This approach aims to reduce network communication data and extend the network’s life cycle. For monitoring area shaped as double squares, a tailored network coverage optimization algorithm is developed. This algorithm quantitatively analyzes both the redundancy of node coverage and the desired coverage levels in critical observation zones, thereby enabling focused attention on these “special” areas.

Define **network coverage** P(S) as the ratio of the area covered by all sensor nodes to the total monitoring area.


$$P\left(S\right)=\left(\sum_{i=1}^{Q}s_{i}\right)/A\left(S\right)$$ (4)

where Q is the number of sensor nodes deployed within the monitoring area, $s_{i}$ is the area covered by the *i*th sensor node $SN_{i}$, and A(S) the total monitoring area size. For a monitoring area shaped as a double square, the expected number of nodes within the key monitoring zone is determined by E(X)=QP(I), where P(I) is the network coverage of the key monitoring area (See Corollary 1 in S1 Appendix for the detailed derivation steps).

For sensor node $SN_{i}$ (i=1,2,⋯,Q), define its **perceptual domain set** ${\mathbb{Z}}_{i}$ as


$${\mathbb{Z}}_{i}=\left\{SN_{k}\right\},0<\left\|SN_{k}-SN_{i}\right\|\leq 2r$$ (5)

where r is the node’s sensing radius. The proportion of nodes within its sensing domain set ${\mathbb{Z}}_{i}$ that cover its own sensing area is defined as the **node coverage redundancy** $\Theta_{i}$.


$$\Theta_{i}=\left(\sum_{SN_{k}\in {\mathbb{Z}}_{i}}s_{i}⋂s_{k}\right)/s_{i}$$ (6)

where $s_{i}\cap s_{k}$ denotes the overlapping area of nodes $SN_{k}$ and $SN_{i}$. It can be proven (Corollary 2 in S1 Appendix) that $\Theta_{i}$ satisfies


$$\Theta_{i}=1-\prod_{j=1}^{SN_{j}\in {\mathbb{Z}}_{i}}\left(1+\frac{\left\|SN_{j}-SN_{i}\right\|\sqrt{4r^{2}-{\left\|SN_{j}-SN_{i}\right\|}^{2}}}{2\pi r^{2}}-\frac{2\arccos\frac{\left\|SN_{j}-SN_{i}\right\|}{2r}}{\pi }\right)$$ (7)

For node $SN_{i}$, if its coverage redundancy $\Theta_{i}\geq P_{\min}\left(S\right)$ ($P_{\min}\left(S\right)$ is the minimum network coverage requirement), it implies that the node’s sensing area is nearly fully covered by other nodes within its ${\mathbb{Z}}_{i}$. Consequently, the node’s status can be switched to dormant.

**Fig 4:**
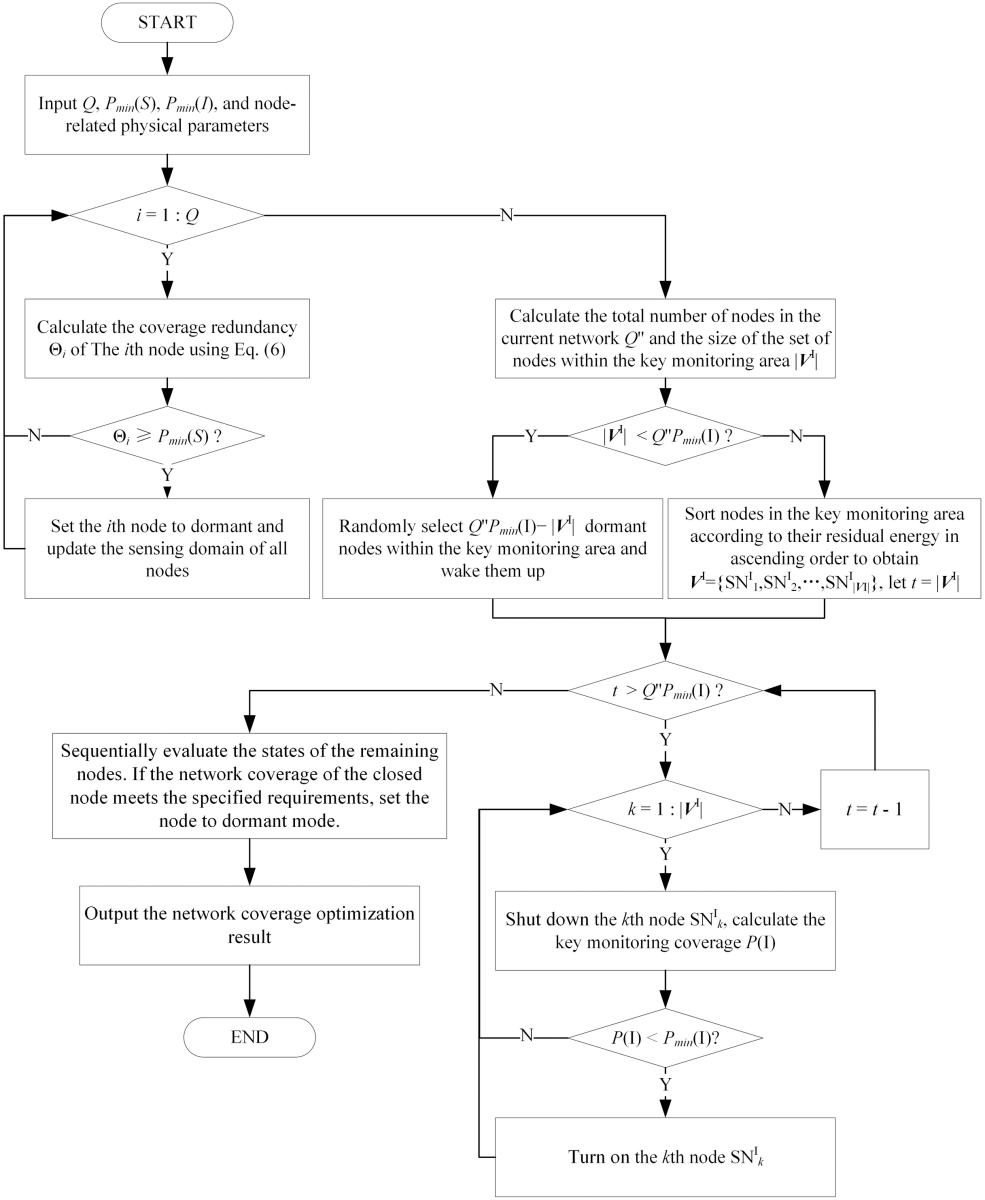
Flowchart of node scheduling.

Based on the expected network coverage of key monitoring area nodes and the node coverage redundancy, a node scheduling strategy is implemented. This strategy allows nodes to alternate between active and dormant states, ensuring comprehensive network coverage while minimizing the number of active nodes. Furthermore, it prioritizes nodes with low residual energy for dormancy, thereby significantly prolonging the network’s life cycle. The pseudo-code of the WSNs node scheduling is presented in Algorithm 1. A clear visual flowchart is illustrated in Fig 4.

**Algorithm 1.** Pseudo-code of WSNs node scheduling

**Inputs:**Q, $P_{\min}\left(S\right)$, $P_{\min}\left(I\right)$, node-related physical parameters.

// Node redundancy determination

1. for i=1:Q

2. Calculate the $SN_{i}$ node coverage redundancy $\Theta_{i}$ according to equation (6);

3. if $\Theta_{i}\geq P_{\min}\left(S\right)$

4. Set $SN_{i}$ to dormant and update the sensing domain of all nodes;

5. end for

//Key monitoring area node status determination

6. Calculate the total number of nodes in the current network $Q^{\prime \prime }$ and the size of the set of nodes within the key monitoring area $\left|V^{I}\right|$;

7. if $\left|V^{I}\right|<Q^{\prime \prime }P_{\min}\left(I\right)$

8. Randomly select $Q^{\prime \prime }P_{\min}\left(I\right)-\left|V^{I}\right|$ sleeping nodes within the key monitoring area and wake them up;

9. else

10. Sort nodes in the key monitoring area according to their residual energy in ascending order to obtain $V^{I}=\left\{SN_{1}^{I},SN_{2}^{I},\cdots ,SN_{\left|V^{I}\right|}^{I}\right\}$, and let $t=\left|V^{I}\right|$;

11. while ($t>Q^{\prime \prime }P_{\min}\left(I\right)$) do

   {

12. for $k=1:\left|V^{I}\right|$

13. Shut down node $SN_{k}^{I}$, calculate the key monitoring coverage P(I), if $P\left(I\right)\geq P_{\min}\left(I\right)$, keep the node $SN_{k}^{I}$ in dormant; else, turn it on.

14. t−1→t

   }

// Non key monitoring area node status determination

15. Sequentially evaluate the states of the remaining nodes. If the network coverage of the closed node meets the specified requirements, set the node to dormant mode.

**Output:**network coverage optimization result.

### Network clustering

Taking the number of time rounds in WSNs as the basic unit, network coverage optimization based on expectations is conducted at the start of each round for densely deployed networks. This process ensures a more uniform deployment of working nodes. For a network with $Q_{w}$ active nodes at a specific operational time round, it is divided into C clusters of equal radius $R_{C}$, where $R_{C}=\eta R$ (R is the communication radius, and η is the maximum number of communication hops within a cluster), C is calculated as


$$Q_{C}=\lambda \pi R_{C}^{2}=\pi \lambda \eta^{2}R^{2}$$ (8)

$$C=\frac{Q_{w}}{Q_{C}}=\frac{Q_{w}}{\pi \lambda \eta^{2}R^{2}}$$ (9)

where $Q_{C}$ is the number of nodes within a cluster and λ is the density of network nodes. From equations (8) and (9), it is evident that a larger number of clusters in the network leads to more inter-cluster communications. Conversely, a smaller number of clusters results in increased intra-cluster communications. Hence, determining an optimal cluster size can effectively balance intra-cluster and inter-cluster communications, thereby reducing the energy consumption associated with network communication.

When the maximum number of intra-cluster communication hops η satisfies equation (10), the network achieves optimal benefits in both intra-cluster and inter-cluster communications (Corollary 3 in S1 Appendix).


$$\eta ={\left(\frac{6\pi \lambda \mu_{1}\mu_{2}R^{2}+9\mu_{1}^{2}}{{\left(2\pi \lambda \mu_{2}R^{2}\right)}^{2}}+\sqrt{\frac{1}{1728}}\right)}^{\frac{1}{3}}+{\left(\frac{6\pi \lambda \mu_{1}\mu_{2}R^{2}-9\mu_{1}^{2}}{{\left(2\pi \lambda \mu_{2}R^{2}\right)}^{2}}+\sqrt{\frac{1}{1728}}\right)}^{\frac{1}{3}}$$ (10)

Here, $\mu_{1}$ and $\mu_{2}$ are the weight factors, typically determined based on historical data to balance intra-cluster/inter-cluster communication overhead.

**Fig 5:**
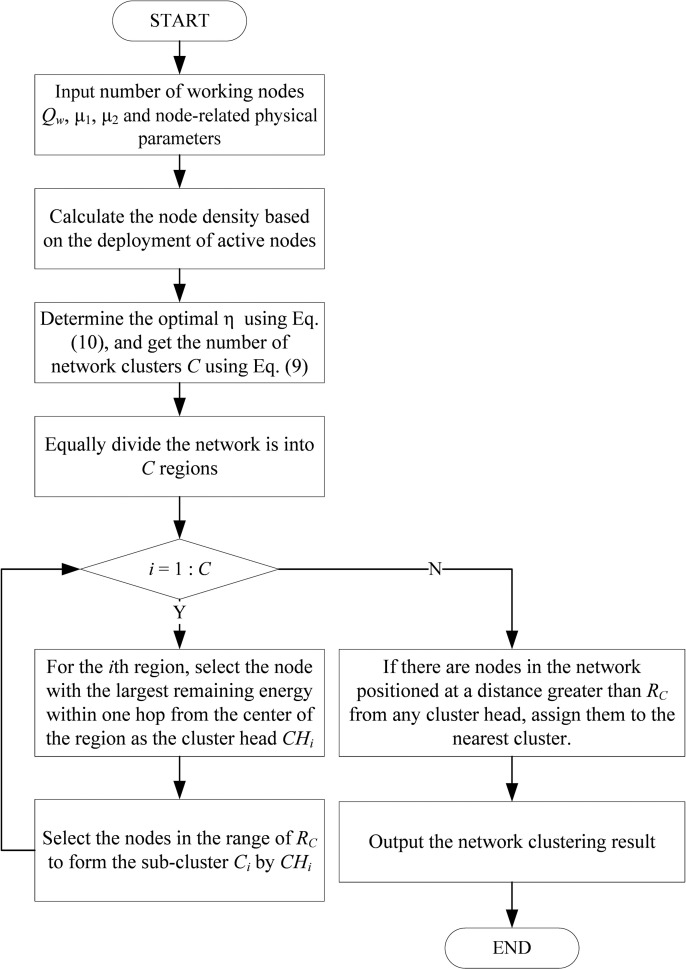
Flowchart of network clustering.

It is evident that the cluster size varies with the density of network nodes, representing a dynamic adaptive adjustment process. As the network operates over an extended period, the number of active nodes gradually diminishes, causing the cluster radius to progressively expand. This adaptation is advantageous in minimizing the energy consumption of network nodes. The pseudo-code of the network clustering is presented in Algorithm 2. A clear visual flowchart is illustrated in Fig 5.

**Algorithm 2.** Pseudo-code of network clustering.

**Inputs:**number of working nodes $Q_{w}$, $\mu_{1}$, $\mu_{2}$ and node-related physical parameters.

**1.** Calculate the node density based on the deployment of active nodes;

**2.** Determine the optimal η according to equation (10), and get the number of network clusters *C* according to equation (9);

//Cluster head selection and node division

**3.** Equally divide the network into C regions;

**4.** for i=1:C

**5.** For the i th region, select the node with the largest remaining energy within one hop from the center of the region as the cluster head $CH_{i}$;

**6.** Select the nodes in the range of $R_{C}$ to form the sub-cluster $C_{i}$ by $CH_{i}$;

**7.** end for

// Individual work node division

**8.** If there are nodes in the network positioned at a distance greater than $R_{C}$ from any cluster head, assign them to the nearest cluster.

**Output:**network clustering result.

### Cluster-based compressive sensing

The advent of CS has revolutionized signal acquisition and data processing, enabling the sensing of high-dimensional signals through uncorrelated observations in low-dimensional spaces [17]. Within a specific cluster in the network, based on the data collection model, the cluster head $CH_{i}$ (i=1,2,⋯,C) employs the measurement matrix $\Phi_{M\times Q_{k}}$ to project the node data within the cluster $X_{Q_{k}\times 1}={\left(f_{1}^{k},\cdots ,f_{Q_{k}}^{k}\right)}^{T}$ yielding $y_{M\times 1}$, and $G_{N^{2}\times 1}$ is K -degree ($K\ll N^{2}$) sparse on the sparse basis $\Psi_{Q_{k}\times N^{2}}$ for $X_{Q_{k}\times 1}$. Here, we have


$$y_{M\times 1}=\Phi_{M\times Q_{k}}\Psi_{Q_{k}\times N^{2}}G_{N^{2}\times 1}=A_{M\times N^{2}}G_{N^{2}\times 1}$$ (11)

According to CS, when $A_{M\times N^{2}}=\Phi_{M\times Q_{k}}\Psi_{Q_{k}\times N^{2}}$ satisfies the restricted isometry property (RIP) condition, $G_{N^{2}\times 1}$ can be sparsely reconstructed by solving an optimization problem, and then $X_{Q_{k}\times 1}={\left(f_{1}^{k},\cdots ,f_{Q_{k}}^{k}\right)}^{T}$ can be obtained. Furthermore, in WSNs, the data compression process is completed at the cluster head $CH_{i}$, while the data reconstruction occurs at the sink node. This approach aligns well with the inherent physical characteristics of WSNs, effectively reducing overall energy consumption within the network. Given the correlation and sparsity inherent in the data collected by WSN nodes, the measurement matrix $\Phi ={\left(\phi_{ij}\right)}_{M\times Q_{k}}$ (usually $M\ll Q_{k}$) is structured as follows


$$\phi_{ij}=\sqrt{s}\times \left\{ \begin{array}{c} 1,p=\frac{1}{2s}\left(1-\frac{K}{N^{2}}\right)\\ 0,p=1-\frac{N^{2}-K}{N^{2}s}\\ -1,p=\frac{1}{2s}\left(1-\frac{K}{N^{2}}\right) \end{array}\right.$$ (12)

where s is denoted as the sparsity of $\Phi_{M\times Q_{k}}$, it can be proved that the measurement matrix $\Phi_{M\times Q_{k}}$ using equation (12) satisfies the RIP condition. From the definition of $\Phi_{M\times Q_{k}}$, it is evident that a smaller s, coupled with higher sparsity K of $G_{N^{2}\times 1}$, results in a denser matrix $\Phi_{M\times Q_{k}}$. This, in turn, suggests an increased need for data communication within the cluster.

### Weak correlation observation matrix

During the operation of the actual WSNs network, link packet loss frequently occurs. If the erroneous link data are not addressed, they can lead to significant structural errors in the final data reconstruction. Observations in WSNs reveal a spatial correlation between nodes [5], which can be expressed in terms of the distance between them: the closer the nodes, the stronger the correlation. Conversely, if the distance between two nodes exceeds 2R, the correlation becomes negligible. Hence, the weak correlation matrix $W_{C}$ is constructed by normalizing the reciprocal of node distance, enabling the estimation and repair of lost data through the spatial correlation of adjacent nodes.


$$W_{C}={\left(w_{ij}\right)}_{Q_{k}\times Q_{k}}=\left\{ \begin{array}{c} 1,i=j\\ \frac{1}{\left\|SN_{i}-SN_{j}\right\|},0<\left\|SN_{k}-SN_{i}\right\|\leq 2R\\ 0,else \end{array}\right.$$ (13)

Normalization to $W_{C}$ is available


$$H_{C}={\left(h_{ij}\right)}_{Q_{k}\times Q_{k}}={\left(\frac{w_{ij}}{\sum w_{ij}}\right)}_{Q_{k}\times Q_{k}}$$ (14)

At this point, the cluster node data is transformed to ${X^{new}}_{Q_{k}\times 1}$


$${X^{new}}_{Q_{k}\times 1}=H_{C}X_{Q_{k}\times 1}$$ (15)

When an error occurs in the node data $f_{i}^{k}$, set the i th column of $H_{C}$ to zero, then a new weak correlation matrix $H_{C}^{1}$ is obtained. This matrix $H_{C}^{1}$ is then used to correct the node data within the cluster, i.e.,


$$\begin{array}{c} {X^{h}}_{Q_{k}\times 1}=\left[\begin{array}{c} {}*20cf_{1,,new}^{k}\\ \begin{array}{c} {}*20c\begin{array}{c} {}*20c\vdots \\ f_{i,new}^{k} \end{array}\\ \vdots \end{array}\\ f_{Q_{k},new}^{k} \end{array}\right]=\left[\begin{array}{c} {}*20cf_{1}^{k}+\cdots +0\times f_{i}^{k}h_{1i}+\cdots +f_{Q_{k}}^{k}h_{1Q_{k}}\\ \begin{array}{c} {}*20c\vdots \\ 0\times f_{i}^{k}+f_{1}^{k}h_{i1}+\cdots +f_{Q_{k}}^{k}h_{iQ_{k}} \end{array}\\ \vdots \\ f_{Q_{k}}^{k}+\cdots +0f_{i}^{k}h_{Q_{k}i}+\cdots +f_{Q_{k-1}}^{k}h_{Q_{k}Q_{k}-1} \end{array}\right]\\ =\left[\begin{array}{cccc} {}*20c1 & \begin{array}{cc} {}*20c\cdots & 0 \end{array} & \cdots & h_{1Q_{k}}\\ \vdots & \begin{array}{cc} {}*20c & \vdots \end{array} & & \vdots \\ \begin{array}{c} {}*20ch_{i1}\\ \vdots \end{array} & \begin{array}{cc} {}*20c\begin{array}{c} {}*20c\cdots \end{array} & \begin{array}{c} {}*20c0\\ \vdots \end{array} \end{array} & \begin{array}{c} {}*20c\cdots \end{array} & \begin{array}{c} {}*20ch_{iQ_{k}}\\ \vdots \end{array}\\ h_{Q_{k}1} & \begin{array}{cc} {}*20c & 0 \end{array} & \cdots & 1 \end{array}\right]\left[\begin{array}{c} {}*20cf_{1}^{k}\\ \begin{array}{c} {}*20c\begin{array}{c} {}*20c\vdots \\ f_{i}^{k} \end{array}\\ \vdots \end{array}\\ f_{Q_{k}}^{k} \end{array}\right]=H_{C}^{1}X_{Q_{k}\times 1} \end{array}$$ (16)

From equation (16), it can be seen that when a link error occurs, the weak correlation matrix leverages the correct data from other nodes to estimate the error-prone node. This process mitigates the impact of the miscommunicating node on the data vectors of nodes within the cluster. Consequently, the CS model is transformed into


$$y_{M\times 1}=\Phi_{M\times Q_{k}}H_{C}^{1}X_{Q_{k}\times 1}=\Phi_{M\times Q_{k}}H_{C}^{1}\Psi_{Q_{k}\times N^{2}}G_{N^{2}\times 1}=A_{M\times N^{2}}^{\prime \prime }G_{N^{2}\times 1}$$ (17)

### CS Reconstruction

To enhance the accuracy of the CS reconstruction algorithm, this paper integrates Sparrow Search Algorithm (SSA) into the sparse signal reconstruction process. Inspired by sparrows’ foraging behavior, SSA employs a discoverer-follower role and vigilance mechanism to balance global exploration and local refinement [18]. This bio-inspired optimization enables high reconstruction accuracy under packet loss, while eliminating the need for sparsity pre-estimation.

Define the objective function of the SSA optimized CS reconstruction algorithm as


$$\min f\left(G\right)={\left\|y-\Phi_{M\times Q_{k}}H_{C}^{1}\Psi_{Q_{k}\times N^{2}}G_{N^{2}\times 1}\right\|}_{2}$$ (18)

Referring to the event source signal vector $G_{N^{2}\times 1}={\left(g_{1},\cdots ,g_{j},\cdots ,g_{N^{2}\times 1}\right)}^{T}$, define the SSA sparrow encoding method as


$$F_{i}\left(x_{j}\right)=x_{j}\left\{ \begin{array}{c} 1,g_{j}\neq 0\\ 0,g_{j}=0 \end{array}\right.j=1,2,\cdots ,N^{2}$$ (19)

where $F_{i}$ is the sparrow individual.

**Fig 6:**
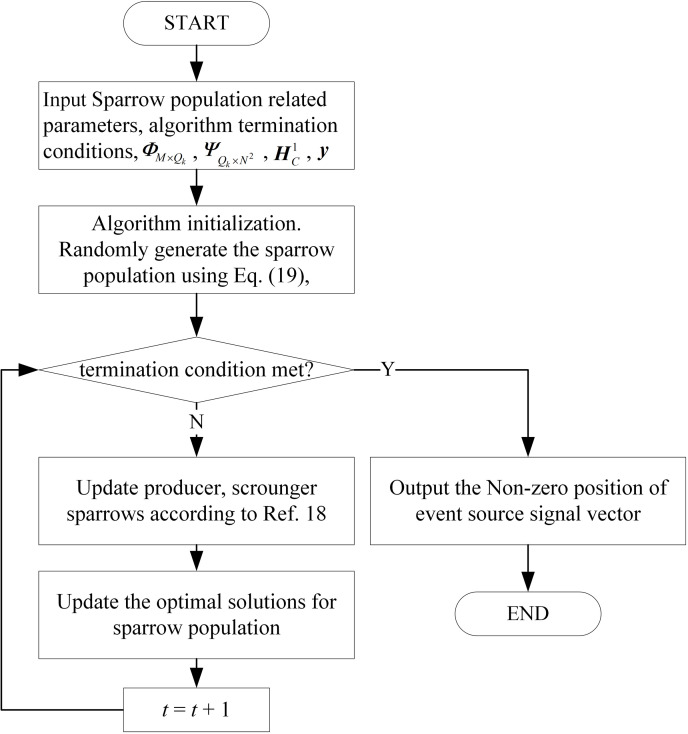
Flowchart of CS reconstruction.

In SSA, 10% to 20% of the total sparrows are assumed to be aware of the danger, safeguarding the whole population. During the initial stage of iterations, SSA is biased for global exploration, the number of the alerters should be as much as possible. Yet in the later iterations of SSA, the algorithm is biased towards local exploitation, the number of alerters should be reduced as much as possible while ensuring the safety of the whole population. Therefore, we can dynamically adjust the ratio of the alerters in accordance with the progress of iteration


$$SD=0.2⬝\cos\left(\frac{\pi }{3}a\right)$$ (20)

where *SD* denotes the percentage of sparrows who perceive the danger, *t* denotes the current number of iterations, *T* is the maximum iterations. The pseudo-code of the CS reconstruction is presented in Algorithm 3. A clear visual flowchart is illustrated in Fig 6.

**Algorithm 3.** Pseudo-code of CS reconstruction.

**Input:** Sparrow population related parameters, algorithm termination conditions; $\Phi_{M\times Q_{k}}$, $\Psi_{Q_{k}\times N^{2}}$, $H_{C}^{1}$, y.

**1.** Algorithm initialization. Randomly generate the sparrow population according to equation (19), t=0;

**2.** hile (termination condition not met)

   {

**3.** Update producer, scrounger sparrows according to reference 18;

**4.** Update the optimal solutions for sparrow population;

**5.** t+1→t;

   }

**Output:**Non-zero position of event source signal vector $G_{N^{2}\times 1}={\left(g_{1},\cdots ,g_{j},\cdots ,g_{N^{2}\times 1}\right)}^{T}$.

The SSA optimized CS reconstruction algorithm is employed to identify the non-zero positions of the event source signal vector. Then, the least squares method is applied to determine the amplitude information of these identified position. Finally, the data collection information of the nodes within the cluster is obtained according to $X_{Q_{k}\times 1}=H_{C}^{1}\Psi_{Q_{k}\times N^{2}}G_{N^{2}\times 1}$, thereby completing the data processing of the nodes in the WSNs.

### Experimental simulation

To verify the effectiveness of the data collection scheme, which is based on expected network coverage and cluster compressive sensing, experimental simulations were conducted on Microsoft Windows 10 equipped with 128GB of RAM and a CPU including the specifications of Intel® Core™ i7-9700 (3.00 GHz), developed with MATLAB 2024a.

The monitoring area is a 100 m × 100 m square area. The key monitoring area is a 30 m × 30 m square area centered within the monitoring area. 300 sensor nodes are densely deployed, and a sink node is deployed outside the key monitoring area. The sensing radius and communication radius of each sensor node is set to 15 m and 30 m respectively. The number of event sources within the monitoring area is unknown. Table 1 lists the key parameters of the data collection scheme, while SSA parameters refer to reference 18. The evaluation indicators include network energy consumption E, delay $T_{d}$, network lifetime $L_{f}$, and signal reconfiguration error. The signal reconfiguration error is evaluated by signal-to-noise ratio SNR and relative error RE.

**Table 1:** Key parameters of the data collection scheme.

Parameter	Value	Description
$P_{\min}(S)$	0.85	The minimum network coverage of the monitoring area
$P_{\min}(I)$	0.9	The minimum network coverage of the key monitoring area
$\mu_{1}$	0.4	The weight factors to calculate the maximum number of intra-cluster communication hops in Eq. (10)
$\mu_{2}$	0.6	
s	80/lg80	The sparsity of the measurement matrix of WSN nodes

### Instance simulation analysis

Using the proposed data collection scheme for instance simulation, experiments were conducted for two scenarios: stable link data collection and packet mis-delivery. Data sampling occurred at regular intervals, with evaluation metrics analyzed separately for each round. Table 2 displays the evaluation results for a single round of data collection.

**Table 2:** Data collection performance for a single round.

	E /J	$T_{d}$ /s	$L_{f}$ /10^3^ s	RE	P(S)	P(I)
stable link	1.4	8.5	2.15	0.1	90%	93%
unstable link	1.7	9.2	1.75	0.12		

As can be seen from Table 2, the cluster-based compressed sensing data collection scheme achieves a signal reconstruction error of only 0.1 in stable network environment. Even in unstable environment, the reconstruction error is merely 0.12. This demonstrates that the scheme can effectively complete data collection with high accuracy.

### Comparative experiments

**Fig 7:**
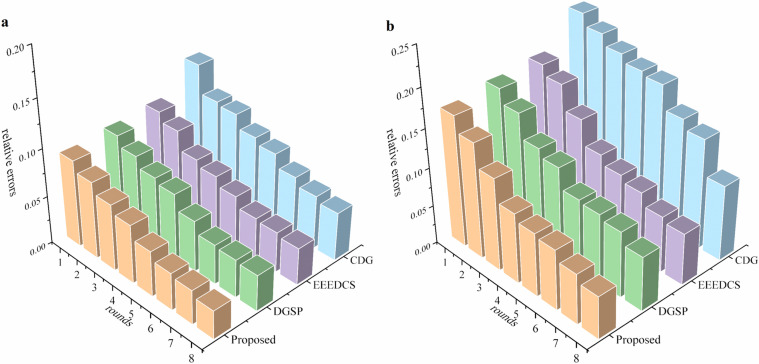
Comparative analysis of relative errors in 4 schemes. **(a) under stable link; (b) under unstable link**.

**Fig 8:**
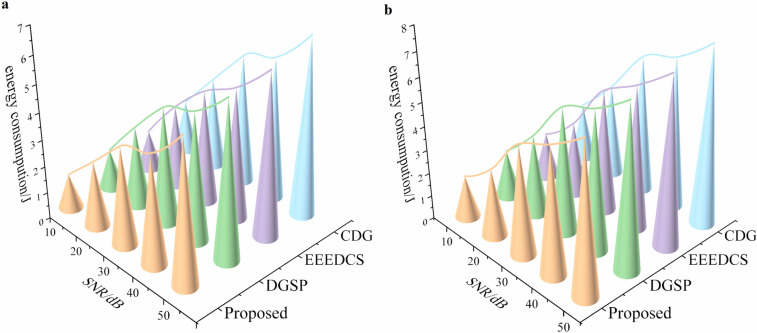
Comparative analysis of energy consumption in 4 schemes. **(a) energy consumption under stable link; (b) energy consumption under unstable link**.

**Fig 9:**
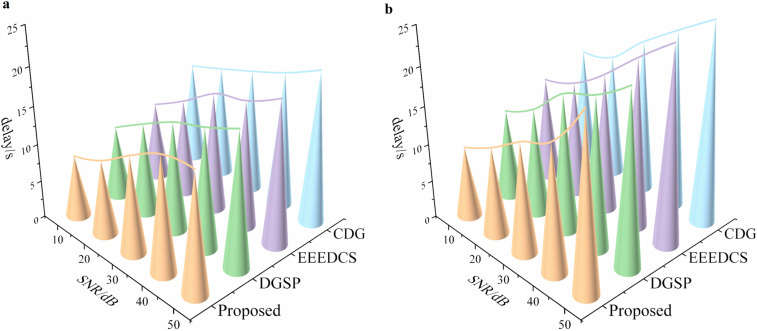
Comparative analysis of delay among 4 schemes. **(a) delay under stable link; (b) delay under unstable link**.

**Fig 10:**
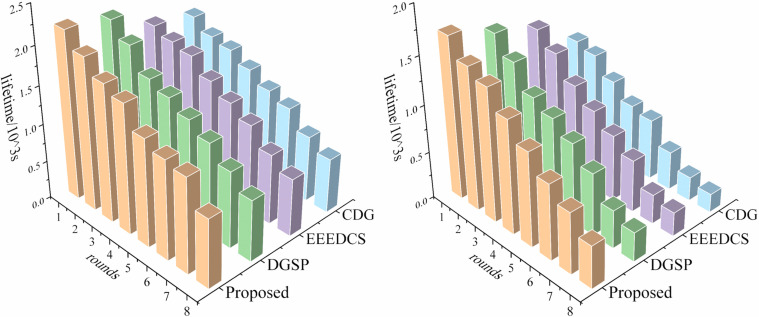
Comparative results of life cycle across 4 schemes. **(a) life cycle under stable link; (b) life cycle under unstable link**.

To further analyze the performance of the data collection scheme presented in this paper, we selected the DGSP scheme from reference 12, the EEEDCS scheme from reference 5, and the classical CDG scheme from reference 19 for comparative analysis, Table 3 provides a concise comparison of these schemes. The DGSP scheme, grounded in compressed sensing theory, does not utilize a clustering strategy; instead, it transmits data using a data tree structure. In contrast, the Proposed method stands out with its dynamic optimization approach for coverage, adaptive clustering strategy, utilization of a weak-correlation matrix in compressive sensing, and spatial correlation correction for robustness handling. We evaluated the performance of these four schemes by conducting experiments and comparing several evaluation metrics in both stable and unstable network environments. The Proposed method demonstrates superior performance in terms of energy efficiency, network lifetime extension, and signal reconstruction accuracy. Its dynamic and adaptive features allow for better optimization of network resources and more effective handling of data transmission challenges, making it highly suitable for wireless sensor network applications. The results of these comparisons are illustrated in Figs 7, 8, 9, and 10, showcasing the differences across various evaluation indicators.

**Table 3:** Comparative analysis of data collection schemes.

Method	Coverage optimization	Clustering strategy	Compressive sensing	Robustness handling
DGSP [12]	None	Static	Sparse projection matrix (RIP-based)	Discard lost data
EEDCS [5]	None	Random clustering	Fixed projection	None
CDG [19]	None	None	Dense matrix	Redundant measurements and joint decoding
Proposed	Dynamic optimization	Adaptive	Weak-correlation matrix	Spatial correlation correction

As shown in Fig 7, with increasing sampling instances, the relative errors of the four algorithms decrease. This occurs because sparse sampling at low iteration counts fails to capture sufficient spatial correlations, leading to reconstruction inaccuracies. However, the proposed scheme outperforms the other three in both stable and unstable networks, especially under packet loss conditions. The proposed scheme achieves a 21% lower relative error than CDG under packet loss. Unlike DGSP and EEEDCS, which discard lost data, our method estimates missing values through spatial correlations, reducing error propagation by leveraging node deployment topology. Furthermore, the integration of SSA employs a discoverer-follower-vigilance mechanism to avoid local optima traps inherent in traditional OMP employed by DGSP. Discoverers guide global exploration, followers refine local solutions, and vigilant agents introduce randomness, collectively improving sparse signal recovery accuracy.

Figs 8 and 9 show that energy consumption and delay increase as the SNR rises among the four schemes. This trend is attributed to the need to increase the number of sampling nodes to enhance SNR, which in turn raises the network’s communication throughput and leads to higher energy consumption and delay. However, at the same SNR level, the proposed scheme consistently outperforms the other three. This is attributed to the adaptive cluster sizing balances intra-cluster computation and inter-cluster communication, avoiding energy hotspots caused by static or random clustering. What’s more, by prioritizing low-energy nodes for dormancy, the proposed scheme maintains coverage with fewer active nodes, directly reducing the transmission overhead and leading to a more balanced network load.

Fig 10 shows that as the number of samples increases, the network’s total energy continuously declines, reducing its lifespan. Nevertheless, the proposed scheme’s life cycle surpasses the other three schemes over time, particularly in unstable environments, with an extension of about 20%. This is mainly due to the introduction of the dynamic coverage optimization, which reduces the number of active nodes to avoid redundant data transmission. Additionally, cluster-based compression distributes the computational load to cluster heads, reducing long-distance multi-hop communication payload.

As the network size increases, the growing number of sensor nodes can enhance the coverage of the network. More nodes provide more comprehensive area coverage and reduce blind spots. But in practical applications, having too much nodes is often unnecessary. Initially, as the number of nodes increases, the latency tends to decrease because data can find shorter paths more quickly. However, once the number of nodes reaches a saturation point, competition for network resources, such as bandwidth, becomes more intense, resulting in a significant increase in latency. Regarding energy consumption, during the early stages of network expansion, the increase in node density allows for a relatively even distribution of energy consumption, leading to a reduction in overall energy usage. However, when the network size becomes excessively large, congestion and increased retransmissions due to node collisions will lead to a rise in energy consumption per node.

### Complexity of the proposed scheme

Our WSN data collection scheme combines expected network coverage optimization with cluster-based compressive sensing. The node activation begins by calculating coverage redundancy through evaluating overlapping areas with neighboring nodes. This process requires Q² computational steps in worst-case scenarios where all nodes exhibit overlapping sensing ranges. Subsequent state determination utilizes the coverage redundancy and the expected coverage value of the key monitoring area to achieve O(Q log Q) efficiency.

The compressive data aggregation operates through C cluster formations, each containing approximately q member nodes. Cluster heads perform projection operations using M-dimensional measurement matrices, requiring CqM operations per sampling interval. Post-compression transmission exhibits linear scalability (O(CM)) as compressed packets route through optimized paths to the sink node. Reconstruction employs SSA, which maintains P candidate solutions through T evolutionary iterations. Each iteration performs N-dimensional vector updates, resulting in O(TPN) computational load for signal recovery.

Memory allocation patterns reveal distinct spatial requirements across operational phases. Node scheduling maintains O(Q) storage for node states and coverage maps. Cluster management demands O(Cq) space for membership records and routing tables. Measurement matrices occupy O(CMq) total storage across all clusters. SSA reconstruction requires O(PN) space for solution population maintenance.

In summary, the time complexity of the scheme proposed in the paper is primarily concentrated in the node scheduling, network clustering, data collection and compression, and data reconstruction. The overall time complexity can be expressed as O(Q^2^ + QlogQ + CqM + CM + TPN). The space complexity is mainly concentrated in storing node information, cluster information, measurement matrices, sparse matrices, and other data, with the overall space complexity being O(Q + Cq + CMq + PN). Key parameters are listed in Table 4.

**Table 4:** Key parameters for time complexity and space complexity.

Parameter	Description
Q	Total sensor nodes
C	Number of clusters
q	Average cluster size
M	Measurement dimensions
N	Sparse signal length
P	SSA population size
T	Evolutionary iterations

## Conclusions

In various practical applications of wireless sensor networks, there is an increasing demand for real-time data monitoring and high accuracy in data collection and reconstruction. To address these needs, this paper introduces a data collection scheme for WSNs that utilizes expected network coverage and cluster compressive sensing. Initially, the scheme employs the K-medoids clustering method to logically cluster the wireless sensor networks, optimizing the distribution of node distances within each cluster. Subsequently, the process involves optimizing network coverage, conducting network clustering, collecting compressed sensing data, and reconstructing sparse signals. Through simulation, a detailed analysis of data collection, network coverage optimization, and network clustering is performed under both stable and unstable link conditions. This analysis confirms that the sparse signals processed through cluster compressive sensing on network nodes yield high reconstruction accuracy. Simulation experiments conducted in both stable and unstable network environments demonstrate these findings.

(1) In this paper, the integration of error data estimation for discrete weak correlation matrices and the sparrow search algorithm into the sparse signal reconstruction process is introduced to achieve precise reconstruction of signals with unknown sparsity. This method demonstrates a robust global search capability, enhancing the overall performance of the algorithm, particularly in unstable network environments. Compared to other data collection schemes, the reconstruction accuracy is significantly improved.
(2) In this paper, we employ network clustering in conjunction with a network coverage optimization mechanism. This approach minimizes the amount of sampling data required from cluster member nodes, as communication occurs solely among nodes within the cluster without necessitating interaction with the sink node. Compared to traditional WSN data collection methods, our scheme significantly reduces energy consumption among cluster member nodes and extends the network’s lifecycle.

Therefore, the data collection scheme proposed in this paper offers a valuable reference for enhancing the practicality of WSNs. Future work could be focused on the following key directions:

(1) Integration of mobile sink nodes with UAV-assisted dynamic path planning to improve coverage flexibility in large-scale deployments;
(2) Security-enhanced compressive sensing through lightweight homomorphic encryption to protect sensitive monitoring data;
(3) Extension to 3D environments, such as underwater or mountainous terrains, to address multi-layer monitoring challenges.

These advancements will further bridge the gap between theoretical frameworks and real-world WSN applications.

## Supporting information

S1 Appendix Appendix: Relevant definitions. (DOCX)

S1 Data Dataset. (DOCX)
