# Supplementary material for: WSNs data acquisition by combining expected network coverage and clustered compressed sensing
Source: PLoS One. 2025 Jun 17;20(6):e0326078. doi: 10.1371/journal.pone.0326078 (PMC12173425; doi:10.1371/journal.pone.0326078)
Supplement: S1 Appendix — (DOCX) [file pone.0326078.s001.docx]

**Appendix: Relevant definitions**

**Corollary 1** For a monitoring area shaped as a double square, the expected number of nodes within the key monitoring zone is determined by $E\left(X\right)=QP\left(I\right)$, where $P\left(I\right)$ is the network coverage of the key monitoring area.

**Proof** If the number of nodes in the key monitoring area I is $k$, then the number of nodes in the non key monitoring area is $Q-k$. Consequently, the variable $X$ satisfies a binomial distribution $\left(Q,P\left(I\right)\right)$, where $P\left(I\right)$ is the network coverage in the key monitoring area, i.e.

$P\left\{X=k\right\}=\left(\begin{matrix} Q \\ k \end{matrix}\right)P^{k}\left(I\right)\left[1-P\left(I\right)\right]^{Q-k}$ (A.1)

Then there is

$\begin{matrix} E\left(X\right)=\sum_{k=1}^{Q} kP\left\{X=k\right\}=\sum_{k=1}^{Q} k\left(\begin{matrix} Q \\ k \end{matrix}\right)P^{k}\left(I\right)\left[1-P\left(I\right)\right]^{Q-k} \\ \begin{matrix} & \end{matrix}=\sum_{k=1}^{Q} \frac{kQ!}{k!\left(Q-k\right)!}P^{k}\left(I\right)\left[1-P\left(I\right)\right]^{Q-k} \\ \begin{matrix} & \end{matrix}=\sum_{k=1}^{Q} \frac{QP\left(I\right)\left(Q-1\right)!}{\left(k-1\right)!\left[\left(\left(Q-1\right)\right)-\left(k-1\right)\right]!}P^{k-1}\left(I\right)\left[1-P\left(I\right)\right]^{\left(Q-1\right)-\left(k-1\right)} \\ \begin{matrix} & \end{matrix}=QP\left(I\right)\sum_{k=1}^{Q} \frac{\left(Q-1\right)!}{\left(k-1\right)!\left[\left(\left(Q-1\right)\right)-\left(k-1\right)\right]!}P^{k-1}\left(I\right)\left[1-P\left(I\right)\right]^{\left(Q-1\right)-\left(k-1\right)} \\ \begin{matrix} & \end{matrix}=QP\left(I\right)\left[P\left(I\right)+1-P\left(I\right)\right]^{Q-1}=QP\left(I\right) \end{matrix}$ (A.2)

**Proof completed.**

**Corollary 2** For a given node $SN_{i}$ ($SN_{i}\in V$), its node coverage redundancy $\Theta_{i}$ satisfies

(A.3)

**Proof** for node $SN_{i}$, select the node $SN_{j}$ within the perceptual domain set . There exists an overlap in the perceptual regions of the two nodes, as illustrated in Fig A1. Denote the location of the two nodes by $A$ and $B$, respectively. Consequently, $AB=\left\|SN_{j}-SN_{i}\right\|$. In $\Delta ABC$, there are

$\angle CAB=\angle CBA=\alpha=\arccos\frac{AB}{2r}$ (A.4)

Let the overlapping part of the perceptual region of two nodes is $s_{i,j}$, then there is

(A.5)

Obviously, the probability that any point in the perceptual region of $SN_{i}$ covered by $SN_{j}$ is

$P_{j}=\frac{s_{i,j}}{\pi r^{2}}=\frac{2\arccos\frac{AB}{2r}}{\pi}-\frac{AB\sqrt{4r^{2}-AB^{2}}}{2\pi r^{2}}$ (A.6)

Therefore, the probability that a point within the sensory region of $SN_{i}$ is covered by a node within is

(A.7)

It can be derived from equation (A.7) that the node coverage redundancy$\Theta_{i}$ of the node $SN_{i}$ is satisfied

(A.8)

**Proof completed.**

**Fig A1. The overlapping of the perceptual regions of the two nodes.**

**Corollary 3** When the maximum number of intra-cluster communication hops $\eta$ satisfies equation (A.9), the network achieves optimal benefits in both intra-cluster and inter-cluster communications.

$\eta=\left(\frac{6\pi\lambda\mu_{1}\mu_{2}R^{2}+9\mu_{1}^{2}}{\left(2\pi\lambda\mu_{2}R^{2}\right)^{2}}+\sqrt{\frac{1}{1728}}\right)^{\frac{1}{3}}+\left(\frac{6\pi\lambda\mu_{1}\mu_{2}R^{2}-9\mu_{1}^{2}}{\left(2\pi\lambda\mu_{2}R^{2}\right)^{2}}+\sqrt{\frac{1}{1728}}\right)^{\frac{1}{3}}$ (A.9)

**Proof** The maximum mathematical description of the intra-cluster and inter-cluster communication benefits of the network is

(A.10)

where is the total number of communication hops within the cluster, $\mu_{1}$ and $\mu_{2}$ are the weight factors. The number of nodes in the cluster that require at least $\beta$ hops to communicate with the cluster head $Q_{\beta}$ is

$Q_{\beta}=\lambda\pi\left(\beta R\right)^{2}=\pi\lambda\beta^{2}R^{2}$ (A.11)

Similarly, the number of nodes that require at least $\beta-1$ hops is $Q_{\beta-1}=\pi\lambda\left(\beta-1\right)^{2}R^{2}$, so exactly the number of nodes that require $\beta$ hops $Q_{\beta}^{'}$ is

$Q_{\beta}^{'}=Q_{\beta}-Q_{\beta-1}=\pi\lambda\beta^{2}R^{2}-\pi\lambda\left(\beta-1\right)^{2}R^{2}=\pi\lambda\left(2\beta-1\right)R^{2}$ (A.12)

The number of node communication hops within the cluster is

(A.13)

The number of communication hops for all sub-cluster intra-cluster nodes is

(A.14)

At this point, equation (A.10) is transformed into

$\max\mu_{1}\frac{Q_{w}}{\pi\lambda\eta^{2}R^{2}}+\mu_{2}Q_{w}\left(\frac{2\eta}{3}-\frac{1}{6\eta}+\frac{1}{2}\right)$ (A.15)

where $\eta$ is the variable function, and the partial derivatives is

$\frac{2\mu_{2}}{3}\eta^{3}+\frac{\mu_{2}}{6}\eta^{2}-\frac{2\mu_{1}}{\gamma\pi R^{2}}=0$ (A.16)

Equation (A.16) is a special univariate cubic equation, which is solved to obtain a unique solution, then there is

$\eta=\left(\frac{6\pi\lambda\mu_{1}\mu_{2}R^{2}+9\mu_{1}^{2}}{\left(2\pi\lambda\mu_{2}R^{2}\right)^{2}}+\sqrt{\frac{1}{1728}}\right)^{\frac{1}{3}}+\left(\frac{6\pi\lambda\mu_{1}\mu_{2}R^{2}-9\mu_{1}^{2}}{\left(2\pi\lambda\mu_{2}R^{2}\right)^{2}}+\sqrt{\frac{1}{1728}}\right)^{\frac{1}{3}}$ (A.17)

**Proof completed.**
