# Supplementary material for: WSNs data acquisition by combining expected network coverage and clustered compressed sensing
Source: PLoS One. 2025 Jun 17;20(6):e0326078. doi: 10.1371/journal.pone.0326078 (PMC12173425; doi:10.1371/journal.pone.0326078)
Supplement: S1 Data — (DOCX) [file pone.0326078.s002.docx]

**Data in Figure 7**

| Contrast Group | Method | rounds | | | | | | | |
| --- | --- | --- | --- | --- | --- | --- | --- | --- | --- |
|  |  | 1 | 2 | 3 | 4 | 5 | 6 | 7 | 8 |
| stable link | CDG | 0.131 | 0.102 | 0.098 | 0.084 | 0.078 | 0.064 | 0.056 | 0.050 |
|  | EEEDCS | 0.100 | 0.091 | 0.071 | 0.065 | 0.055 | 0.044 | 0.042 | 0.039 |
|  | DGSP | 0.096 | 0.085 | 0.074 | 0.069 | 0.053 | 0.041 | 0.039 | 0.037 |
|  | Proposed | 0.091 | 0.080 | 0.069 | 0.061 | 0.047 | 0.039 | 0.035 | 0.030 |
| unstable link | CDG | 0.229 | 0.214 | 0.200 | 0.190 | 0.185 | 0.155 | 0.143 | 0.098 |
|  | EEEDCS | 0.186 | 0.173 | 0.141 | 0.109 | 0.097 | 0.089 | 0.073 | 0.067 |
|  | DGSP | 0.179 | 0.161 | 0.130 | 0.120 | 0.094 | 0.091 | 0.085 | 0.070 |
|  | Proposed | 0.170 | 0.150 | 0.120 | 0.091 | 0.082 | 0.078 | 0.065 | 0.055 |

**Data in Figure 8**

| Contrast Group | Method | SNR/dB | | | | |
| --- | --- | --- | --- | --- | --- | --- |
|  |  | 10 | 20 | 30 | 40 | 50 |
| stable link | CDG | 1.940 | 3.395 | 4.820 | 5.280 | 6.555 |
|  | EEEDCS | 1.473 | 3.038 | 4.189 | 4.880 | 6.034 |
|  | DGSP | 1.473 | 2.984 | 4.304 | 4.765 | 5.811 |
|  | Proposed | 1.307 | 2.458 | 3.675 | 4.074 | 5.335 |
| unstable link | CDG | 2.415 | 3.887 | 5.769 | 6.343 | 7.462 |
|  | EEEDCS | 1.908 | 3.086 | 5.142 | 5.987 | 7.083 |
|  | DGSP | 2.014 | 3.406 | 5.275 | 5.882 | 6.877 |
|  | Proposed | 1.801 | 2.873 | 4.659 | 5.362 | 6.343 |

**Data in Figure 9**

| Contrast Group | Method | SNR/dB | | | | |
| --- | --- | --- | --- | --- | --- | --- |
|  |  | 10 | 20 | 30 | 40 | 50 |
| stable link | CDG | 12.971 | 14.463 | 16.051 | 17.778 | 20.039 |
|  | EEEDCS | 10.019 | 12.521 | 15.534 | 16.699 | 19.029 |
|  | DGSP | 9.398 | 12.039 | 14.457 | 16.000 | 18.019 |
|  | Proposed | 8.233 | 9.948 | 12.934 | 15.469 | 16.039 |
| unstable link | CDG | 14.938 | 16.013 | 19.878 | 23.011 | 26.003 |
|  | EEEDCS | 13.431 | 14.910 | 17.998 | 21.937 | 25.466 |
|  | DGSP | 11.658 | 13.969 | 17.998 | 19.878 | 22.994 |
|  | Proposed | 9.402 | 11.644 | 14.670 | 16.923 | 22.994 |

**Data in Figure 10**

| Contrast Group | Method | rounds | | | | | | | |
| --- | --- | --- | --- | --- | --- | --- | --- | --- | --- |
|  |  | 1 | 2 | 3 | 4 | 5 | 6 | 7 | 8 |
| stable link | CDG | 1.701 | 1.552 | 1.493 | 1.356 | 1.208 | 1.107 | 0.869 | 0.721 |
|  | EEEDCS | 1.802 | 1.713 | 1.653 | 1.457 | 1.309 | 1.160 | 0.923 | 0.762 |
|  | DGSP | 2.099 | 1.903 | 1.606 | 1.505 | 1.368 | 1.220 | 1.012 | 0.810 |
|  | Proposed | 2.200 | 1.998 | 1.802 | 1.701 | 1.416 | 1.309 | 1.255 | 0.911 |
| unstable link | CDG | 1.097 | 1.040 | 0.852 | 0.693 | 0.644 | 0.409 | 0.256 | 0.199 |
|  | EEEDCS | 1.403 | 1.250 | 1.011 | 0.852 | 0.701 | 0.556 | 0.313 | 0.256 |
|  | DGSP | 1.545 | 1.352 | 1.097 | 0.994 | 0.852 | 0.663 | 0.406 | 0.313 |
|  | Proposed | 1.705 | 1.506 | 1.409 | 1.205 | 1.003 | 0.807 | 0.651 | 0.455 |
